# Bulgarian Marine and Freshwater Fishes as a Source of Fat-Soluble Vitamins for a Healthy Human Diet

**DOI:** 10.3390/foods2030332

**Published:** 2013-07-19

**Authors:** Mona Stancheva, Diana A. Dobreva

**Affiliations:** Department of Chemistry, Faculty of Pharmacy, Medical University of Varna, 55 Marin Drinov str., Varna, 9000, Bulgaria; E-Mail: mona_stancheva@abv.bg

**Keywords:** Oncorhynchus mykiss, Cyprinus carpio, Mugil cephalus, Sarda sarda, fat soluble vitamins, HPLC/UV/FL

## Abstract

The aim of the present study evaluates the fat-soluble vitamins all-*trans* retinol (vitamin A), cholecalciferol (vitamin D_3_) and α-tocopherol (vitamin E) content in the fresh edible tissue of Bulgarian fish species: marine—grey mullet (*Mugil cephalus*) and bonito (*Sarda sarda*), and freshwater—rainbow trout (*Oncorhynchus mykiss*) and common carp (*Cyprinus carpio*). The sample preparation procedure includes alkaline saponification, followed by liquid-liquid extraction with *n*-hexane. All-*trans* retinol, cholecalciferol and α-tocopherol were analyzed simultaneously using RP-HPLC\UV\FL system with analytical column C18 ODS2 Hypersil™. The fat soluble vitamins content (μg per 100 g wet weight) in the fresh edible fish tissue of analyzed fishes are in the ranges: vitamin A from 2.7 ± 0.4 to 37.5 ± 3.4 μg/100 g ww; vitamin D_3_ from 1.1 ± 0.1 to 11.4 ± 0.6 μg/100 g ww; vitamin E from 121.4 ± 9.6 to 1274.2 ± 44.1 μg/100 g ww. Three fat-soluble vitamins occur in higher amounts in rainbow trout and grey mullet species. According to recommended daily intake (RDI), they are a good source of cholecalciferol.

## 1. Introduction

Fish is an essential source of both macronutrients—proteins and fats, and micronutrients—vitamins and minerals. Therefore, fish consumption is an important component of a balanced human diet [1].

Fat-soluble vitamins are essential nutrients in important biological processes in the human body. Vitamin A, also called retinol, takes place in photoreception and regulates gene expression and cell division, bone growth, teeth development, reproduction, *etc*. Vitamin D_3_ (cholecalciferol) promotes and enhances the absorption and metabolism of calcium and phosphorus in our body. α-Tocopherol is the vitamin E compound with the highest biological activity, which acts as an antioxidant, protecting membrane structures, essential fatty acids, and vitamin A from oxidation [2].

The American Heart Association and FAO/WHO recommend at least two fish servings per week [3,4]. In Bulgaria, the consumption of fish is very low (4.5 kg annual per capita) compared to the average European levels (23 kg annual per capita) [5]. Considering these facts, it is necessary to take into account the importance of consuming fish rich in fat-soluble vitamins and other nutrients.

Fish production in Bulgaria relies on two main sources—commercial fishing and aquaculture. Catches in the Black Sea account for approximately 77.3% of the total fish production in Bulgaria. Aquaculture production (including fresh water fish farming and marine farming of fish and mussels) accounts for approximately 13.8% of total fish production. The main aquaculture fish species in Bulgaria are rainbow trout (*Oncorhynchus mykiss*) and common carp (*Cyprinus carpio*) [6].

The origin of rainbow trout is North America, where it inhabits cold and clear freshwater ponds. Trout is a predator with a varied diet; it eats almost everything [7,8]. The common carp is freshwater omnivorous fish of the family Cyprinidae, which inhabits warm, slow water pools [7,8].

Some marine fish species have a migratory character and others do not migrate. The grey mullet (*Mugil cephalus*) and bonito (*Sarda sarda*) represent the second group and are a subject of perennial fishing of the Black Sea. Grey mullets widely occur in coastal and brackish waters of all temperate and tropical seas. They also frequently occur in rivers and may be found long distances upstream. The grey mullets consume mainly on zooplankton, dead plant matter and detritus [9].

The bonito fish live in open waters. They are predators, feeding primarily on small fishes like anchovy, horse mackerel, young mackerel, sprat, *etc*. For this reason, the bonito is considerably important in the trophic interrelations among pelagic fishes [8].

The goal of the present study was to determine and to compare the fat-soluble vitamins A, D_3_ and E content in raw edible tissue of farmed freshwater rainbow trout and common carp, and Black sea grey mullet and bonito. It also aimed at evaluating the contribution of the analyzed vitamins concerning recommended daily intake (RDI) in Bulgaria.

## 2. Experimental Section

### 2.1. Fish Species

Samples of four fish species were purchased from Varna fish market (Varna, Bulgaira) in spring 2011. Fishes were transported to the laboratory in ice box. They were immediately frozen and stored in a freezer at −20 °C. Prior to analysis, the frozen samples were thawed at 4 °C, for 12 h. The edible tissue was filleted with the skin. Five to nine specimens of each fish species were used as raw material for vitamin analysis. Biometrical and biological characteristics of fishes were determined and noted in Table 1.

**Table 1:** Biometrical and biological characteristics of analyzed fishes.

Fish species	Mean total weight (g ± SD)	Mean total length (cm ± SD)	Habitat	Food habits
Rainbow trout ( *n* = 9)	342.0 ± 24.0	28.0 ± 2.0	Pelagic	Omnivorous
Common carp ( *n* = 5)	1464.0 ± 159.5	55.0 ± 4.0	Demersal	Omnivorous
Grey mullet ( *n* = 9)	293.0 ± 10.5	35.0 ± 2.5	Pelagic	Herbivorous
Bonito ( *n* = 7)	422.0 ± 5.5	40.0 ± 1.5	Pelagic	Carnivorous

### 2.2. Standards and Reagents

Standard used for vitamins determination were purchased from Fluka (all-*trans*-retinol; Fluka, Taufkirchen, Germany) and from Sigma-Aldrich (cholecalciferol, α-tocopherol, l-ascorbic acid; Sigma-Aldrich, Taufkirchen, Germany). HPLC-grade reagents were also from Sigma-Aldrich, Taufkirchen, Germany.

### 2.3. Sample Preparation

Prior to analysis, fish specimens were defrosted and the head, tail, fins, and viscera were removed. Fishes were filleted and homogenized, and used as raw material for the preparation of random tissue samples. The sample preparation was performed using the method of Dobreva *et al.* [10].

An aliquot of the homogenized sample (1000 g) was weighed into a glass tube with a screw cap. Two milliliters (2 mL) of 1% methanolic solution of l-ascorbic acid and 5 mL of 1 M methanolic solution of potassium hydroxide were added. The six parallel samples of edible fish tissue were prepared and subjected to saponification at 80 °C for 20 min. 

The fat-soluble vitamins from the hydrolysate were extracted three times with *n*-hexane (2 mL) by vortexing for 1 min. The combined extracts were evaporated to dryness under gentle stream of nitrogen. Finally, the dry residue of the fat-soluble vitamins fraction was reconstituted in 200 µL methanol and 20 µL aliquot was injected in the HPLC column.

### 2.4. HPLC Conditions

HPLC system (Thermo Scientific Spectra SYSTEM, Thermo Fisher Scientific Inc., Waltham, MA, USA) equipped with RP analytical column ODS2 Hypersil™ 250 × 4.6 mm, 5 μm (Thermo Fisher Scientific Inc., Waltham, MA, USA) was used. The mobile phase was composed of 97:3 = MeOH:H_2_O with a flow rate 1.0 mL/min. The qualitative analysis was performed by comparing the retention times of the mixed methanolic standard solutions of all-*trans*-retinol, cholecalciferol and α-tocopherol. Retinol and cholecalciferol were monitored by UV detection at λ_max_ = 325 nm and λ_max_ = 265 nm, respectively. α-Tocopherol was detected by fluorescence at λ_ex_ = 288 nm and λ_em_ = 332 nm. The measurement was done by the method of external calibration [10].

### 2.5. Statistical Analysis

All samples were analyzed in triplicate. Statistical analysis was done by using Graph Pad Prism 5 software (Thermo Fisher Scientific Inc., Waltham, MA, USA). The results were expressed as average and standard deviation (mean ± SD). The amounts of vitamins were presented as µg per 100 g wet weight (µg/100 g ww).

Column statistics was used for calculation of the means, standard deviations, and the coefficients of variation. One way ANOVA was used to evaluate the differences between the means. Statistical significance was indicated at *p* < 0.05.

## 3. Results and Discussion

Our study established wide differences in retinol, cholecalciferol and α-tocopherol contents among fish species. The vitamin content of all analyzed fishes is shown in Table 2 as µg/100 g ww.

**Table 2:** .vitamin contents in raw fish tissue, µg/100 g ww (mean ± SD).

Analyte	all-*trans*-Retinol	Cholecalciferol	α-Tocopherol
Rainbow trout ^a^	27.0 ± 1.4	11.4 ± 0.6	1112.2 ± 40.3
Common carp ^a^	2.7 ± 0.4	1.1 ± 0.1	617.9 ± 26.6
Grey mullet	37.5 ± 3.4	9.4 ± 1.0	1274.2 ± 44.1
Bonito	32.8 ± 1.4	3.0 ± 0.3	121.4 ± 0.6

^a^ Aquaculture fish.

Vitamin A content ranged from 2.7 to 37.5 µg/100 g ww, with statistically significant differences among all species analyzed (*p* < 0.05). The highest values were found in the grey mullet fillets, while the lowest amount of retinol was observed in common carp raw fillets. Grey mullet and bonito presented similar (*p* > 0.05) analyte values. In all other cases, the quantities are statistically distinguishable (*p* < 0.05). Atanasov *at al.* [11] presented the vitamin A contents in the common carp and rainbow trout in IU (30 IU and 280 IU vitamin A, respectively), which correspond to 9 and 84 µg/100 g ww—these values are in agreement with ours.

The rainbow trout and grey mullet fillets have similar values for cholecalciferol—11.4 and 9.4 µg/100 g ww, respectively (*p* < 0.05). Also similar but significantly lower are the values of vitamin D_3_ of carp (1.1 µg/100 g ww) and bonito (3.0 µg/100 g ww) (*p* < 0.05). Оstermeyer and Schmidt have shown the vitamin D_3_ content in the wet fillets of rainbow trout and common carp to be7.2 and 0.98 µg/100 g ww, respectively, which is in the same order as our data [12].

The amounts of vitamin E in trout (1112.2 µg/100 g ww) and mullet fillets (1274.2 µg/100 g ww) were close, while common carp had half that value (617.9 µg/100 g ww). The lowest amount of α-tocopherol was observed in bonito’s raw tissue (121.4 µg/100 g ww). Ahmadnia *et al*. [13] present data of vitamin E content in raw fillets on grey mullet (1250.0 µg/100 g ww) and common carp (190.0 µg/100 g ww) which are very similar to the data collected by us.

The contents of retinol and α-tocopherol, analyzed in raw edible fish tissue of marine grey mullet and bonito, are of the same magnitude to that in other Black Sea fishes, studied by us [14]. Grey mullet shows highest vitamins A and E and high vitamin D_3_ content. This is probably due to the high levels of these nutrients in its food—algae and plankton.

The amounts of all-*trans* retinol, cholecalciferol and α-tocopherol were compared with the recommended daily intake (RDI) adopted in Bulgaria [15]. The quantities of fat-soluble vitamins (provided by 100 g fish fillet) as a percentage of the DRI are given in the Table 3.

**Table 3:** Percentage contribution of fat-soluble vitamins from analyzed fish.

Analyte	Rainbow trout	Common carp	Grey mullet	Bonito	Average daily intake
all-*trans*-Retinol	3.6%	0.4%	5.0%	4.4%	750 μg *
Cholecalciferol	228.0%	22.0%	188.0%	60.0%	5 μg
α-Tocopherol	7.4%	4.1%	8.5%	0.8%	15 mg

* Average value of the recommended daily intake for adults (male and female).

Bulgarian dietary standards for average daily intake of fat-soluble vitamins are close to those adopted in the European Union [15,16]. An exception is the recommendation for the daily intake of vitamin D_3_ (5 μg for adults in our country whilst the recommendation of the European Union is 10 μg [16]).

According to the Bulgarian dietary standards, analyzed fishes provide low percentage of DRI for retinol (0.4%–5.0%) and α-tocopherol (0.8%–8.5%). The lowest amount of vitamins A and E, were shown by common carp edible tissue (0.4% RDI of retinol) and bonito’s raw fillets (0.8% RDI of α-tocopherol).

The studied fish fillets present considerable amounts of vitamin D_3_ (22.0%–228.0%). Our results show that 100 g raw fish fillet of rainbow trout and grey mullet provide about 228% and 188% vitamin D_3_ in terms of the average daily intake, respectively.

## 4. Conclusions

Two freshwater and two marine fishes were analyzed with the aim to evaluate all-*trans* retinol, cholecalciferol and α-tocopherol contents in relation to their nutritional value and recommended daily intake of vitamins.

The rainbow trout and the grey mullet provide considerable amounts of vitamin D_3_—a quantity almost twice as high as that of the RDI.

Among the studied fish species, the grey mullet has some of the highest and most balanced amounts of the three vitamins.
